# Complete Genome Sequences of Bovine Parainfluenza Virus Type 3 Strain BN-1 and Vaccine Strain BN-CE

**DOI:** 10.1128/genomeA.00247-12

**Published:** 2013-02-21

**Authors:** Takashi Ohkura, Takehiro Kokuho, Misako Konishi, Ken-ichiro Kameyama, Kaoru Takeuchi

**Affiliations:** a Department of Infection Biology, Division of Biomedical Science, Faculty of Medicine, University of Tsukuba, Tsukuba, Ibaraki, Japan; b Biologics Production, Center for Animal Disease Control and Prevention, National Institute of Animal Health, Tsukuba, Ibaraki, Japan; c Viral Disease and Epidemiology Research Division, National Institute of Animal Health, Tsukuba, Ibaraki, Japan

## Abstract

Bovine parainfluenza virus type 3 (BPIV3) is associated with upper respiratory disease in cattle in many countries. Here, we report the complete genome sequences of the BPIV3 BN-1 strain, isolated from cattle in Japan, and the BN-CE vaccine strain, derived from the BN-1 strain by passages in chicken embryo fibroblasts.

## GENOME ANNOUNCEMENT

Bovine parainfluenza virus type 3 (BPIV3) is an enveloped, single-stranded, negative-sense RNA virus belonging to the *Respirovirus* genus of the *Paramyxoviridae* family. BPIV3 is one of the most important viruses associated with the bovine respiratory disease complex (BRDC) in cattle. Complete genome sequences of BPIV3 have been determined previously, and isolated BPIV3 viruses were classified as genotypes A, B, and C (1–5). The BN-1 strain was isolated from cattle in Japan (6) and attenuated by serial cell culture passages in chicken embryo fibroblasts (CEFs), resulting in the BN-CE strain. The attenuated BN-CE strain has been used as a live vaccine for cattle in Japan. In this work, we determined the complete genome sequences of the BN-1 and BN-CE strains. The BN-1 and BN-CE strains were propagated in Madin-Darby bovine kidney cells and CEFs, respectively, and total RNAs were extracted from the virus-infected cells. Three overlapping cDNA fragments spanning the entire lengths of the genomes were synthesized by reverse transcription-PCR. The 5′ termini of the negative- and positive-sense genomes were reverse-transcribed and amplified using a 5′-Full rapid amplification of cDNA ends (RACE) core set (TaKaRa). The complete genomes of the BN-1 and BN-CE strains were each 15,480 bp, which was identical to the size of the 910N strain genome (accession no. D84095) but was 24 nucleotides longer than the Kansas/15626/84 strain genome (accession no. AF178654). Phylogenetic analysis classified both the BN-1 and BN-CE strains into genotype A. When the complete genome sequence of the BN-CE strain was compared with that of the parental BN-1 strain, six nucleotide mutations were detected in the BN-CE genome: at nucleotide position 23 (T to C) in the leader sequence; at position 1942 (A to G) in the P gene; at position 5951 (C to A) in the F gene; and at positions 9822 (T to A), 13031 (T to C), and 13550 (A to G) in the L gene. Amino acid changes were also identified, including a leucine-to-isoleucine change at position 287 in the F gene and a cysteine-to-serine change at position 387 in the L gene. Mixed peaks in the sequence analysis were identified at nucleotide positions 24 (T or C), 5770 (A or C), 7400 (C or T), and 14193 (T or C) in the genome of the BN-1 strain and at positions 34 (T or G), 45 (A or T), 4736 (A or C), 4931 (G or A), 7400 (C or T), 7511 (A or G), 14390 (G or T), and 15019 (G or A) in the genome of BN-CE; this indicates that the bulk of both the BN-1 and BN-CE virus stocks contained a mixed virus population. A comparison of the complete sequences of the wild-type BPIV3 strain and vaccine strains would be valuable for understanding the attenuation mechanism of BPIV3.

### Nucleotide sequence accession numbers.

The genome sequences of the BN-1 and BN-CE strains have been submitted to GenBank under the accession no. AB770484 and AB770485, respectively.
